# Social Communication and pragmatic skills of children with Autism Spectrum Disorder and Developmental Language Disorder

**DOI:** 10.1590/2317-1782/20212021075

**Published:** 2021-12-17

**Authors:** Simone Vasconcelos Rocha Hage, Lidiane Yumi Sawasaki, Yvette Hyter, Fernanda Dreux Miranda Fernandes

**Affiliations:** 1 Departamento de Fonoaudiologia, Faculdade de Odontologia de Bauru – FOB, Universidade de São Paulo – USP, Bauru (SP), Brasil.; 2 Communication Sciences and Disorders, Western Michigan University, Kalamasoo (MI), Estados Unidos da America.; 3 Departamento de Fisioterapia, Fonoaudiologia e Terapia Ocupacional, Faculdade de Medicina – FM, Universidade de São Paulo – USP, São Paulo (SP), Brasil.

**Keywords:** Language Tests, Child Language, Child Development, Speech-Language and Hearing Sciences, Social Communication, Cultural Competence, Communication, Testes de Linguagem, Linguagem Infantil, Desenvolvimento Infantil, Fonoaudiologia, Comunicação Social, Competência Cultural, Comunicação

## Abstract

**Purpose:**

to assess the pragmatic and social communicative abilities of children with Typical Language Development (TLD), Autism Spectrum disorder (ASD) and Developmental Language Disorder (DLD).

**Methods:**

Participants were 40 parents and 29 teachers of 40 children ages between 3 and 6 years. Ten children had DLD, ten had ASD and 20 had typical development. All participants answered to the questionnaire of the “Assessment of Pragmatic Language and Social Communication – APLSC – parent and professional reports – beta research version. Data were submitted to statistical analysis.

**Results:**

The assessment tool was useful in identifying the difference in performance of children with different social communicative profiles.

**Conclusion:**

Children with ASD presented social and pragmatic impairments that were more significant than those presented by children with DLD. However, both children with ASD and with DLD presented more social pragmatic difficulties than children with TLD.

## RESUMO

**Objetivo:**

investigar as habilidades pragmáticas e de comunicação social de crianças com Desenvolvimento Típico de Linguagem (DTL), Transtorno do Espectro do Autismo (TEA) e Distúrbio do Desenvolvimento da Linguagem (DDL).

**Método:**

Participaram 40 pais e 29 professores de 40 crianças com idades entre 3 e 6 anos. Dez crianças tinham DDL, 10 tinham TEA e 20 tinham DTL. Todos os participantes responderam ao questionário “Assessment of Pragmatic Language and Social Communication”(APLSC – Avaliação de linguagem pragmática e comunicação social) – relatórios de pais e profissionais – versão beta. Os dados foram submetidos a análise estatística.

**Resultados:**

O instrumento de avaliação foi útil na identificação das diferenças no desempenho de crianças com diferentes perfis de comunicação social.

**Conclusão:**

Crianças com TEA apresentaram dificuldades pragmáticas e sociais mais significativos do que as crianças com DDL. Entretanto, tanto as crianças com DDL quanto as com TEA apresentaram mais dificuldades comunicativas e sociais do que as crianças com DTL.

## INTRODUCTION

Language development derives from the need to communicate with other people. Language is usually the child`s first socialization experience, mediated by the parents during every-day activities^(1)^. When a child`s language is not functional and interferes with his/her social adaptation, a pragmatic disorder may be observed. Pragmatic disorders may result in different communication symptoms. In some cases, as in the Autism Spectrum Disorders (ASD), the communication impairments go beyond the social communication and affect the abilities to maintain relationships and to show interest in various topics. When severe difficulties in verbal receptive and expressive language occur but no intellectual deficit is observed, the child should be assessed to verify if there is a Developmental Language Disorder (DLD).

ASD’s main features refer to persistent deficit in interaction and social communication, including social reciprocity and verbal communication behavior used in social interactions and in abilities to develop and maintain social relationships. Besides the deficits in social communication, according to the DSM-5 criteria^(2)^, the diagnosis of ASD also includes restricted and repetitive behavioral patterns.. Verbal and non-verbal social communication features vary depending on age, cognitive and linguistic developmental level. Several symptoms can be observed, from total absence of speech, to a mild language delay; deficits in receptive language and echolalia^(2)^. Even when formal language abilities are intact, the use of language to social reciprocal communication is impaired in ASD^(3)^.

Children with ASD usually present appropriate syntactic abilities associated with poor semantic performance^(4,5)^. Deficits in the pragmatic components of language are the central feature of ASD^(6,7)^.

Children can also present language development disorders that are not associated with social reciprocal difficulties, cognitive deficits or hearing loss. The diagnosis of Developmental Language Disorders (DLD) implies these characteristics and invariably includes some pragmatic language disorder^(8)^.

DLD comprises a group of heterogeneous language differences that become more evident with development. It is a persistent disorder that cannot be explained by cognitive, sensorial or motor deficits, brain damage, social-affective deprivation or psychopathological disorders^(9)^. The linguistic features of DLD are varied and changeable, depending on the child`s development and on the complexity of the disorder.

The characteristics of individuals with DLD may include some, but not necessarily all, of the following features: speech onset after the second year of life; immature or inaccurate production of speech sounds, especially in preschoolers; simplified grammatical structures; lack of age-appropriate verbal markers; restricted expressive and receptive vocabulary; poor short term memory and difficulties in understanding complex language or faster rate of speech^(9)^.

There are a few hypotheses about the linguistic deficits in DLD^(9)^. One of them suggests that the child`s linguistic competence is intact, but there is a difficulty in transforming information in speech sign^(9)^; therefore, the poor performance in auditory processing may be a risk factor since it can interfere with the abilities to discriminate speech sounds. Other studies point out that there may be innate neurological deficits in linguistic processing mechanisms and that this impairment may be associated with limited information processing by verbal memory^(10)^. Recently, another study^(11)^ suggested that there is a memory deficit specifically related to lexical processing that may result in comprehension deficits in children with DLD.

Other neurodevelopmental disorders may result in pragmatic deficits, but ASD and DLD are significant representations of communication disorders that affect in larger or smaller level the social communicative and pragmatic aspects of language^(12)^. It is important to consider that despite the different characteristics of children with one of these diagnoses, there is evidence of a number of children that receive the DLD diagnosis but develop ASD symptoms during their teenage years^(13)^.

Even knowing that social communicative abilities are more affected in children with ASD than in children with DLD it may be useful to use a specific tool to assess these differences and how they are associated with the data about children with typical language development (TLD)^(14)^. Previous studies have shown that parents can be reliable informants about the child’s communicative abilities^(15,16)^. This information may help designing individual intervention programs based on specific profiles of abilities and inabilities.

The aim of this study was to assess the pragmatic and social communicative abilities of children with TLD, ASD and DLD.

## METHODS

This study was approved by the Research in Ethics Committee of the Higher Education Institution where it was carried-out (# 58337716.9.1001.5417). The parents or caregivers of all participants, as well as the professionals participating in the study, signed the approved consent form.

### Participants

Participants were 40 parents and 29 teachers of 40 children ages between 3 years, 6 months and 6 years, 11 months. Participants of the Comparative Group were paired with the Experimental Group according to the children’s ages, type of school and sex. The distribution of the participants is explained bellow in Chart 1:

**Chart 1:** Distribution of the participants

Experimental Group (EG)	Comparative Group (CG)
10 parents of children with ASD	10 parents of children with TLD
(EG – Parents ASD)	(CG – Parents TLD)
10 parents of children with DLD	10 parents of children with TLD
(EG – Parents DLD)	(CG – Parents TLD)
4 teachers of children with ASD	15 teachers of children with TLD
(EG – Teachers ASD)	(CG – Teacher TLD)
10 teachers of children with DLD	
(EG- Teachers ASD)	

**Caption:** EG = experimental group; CG = control group; ASD = autism spectrum disorders; DLD = developmental language disorders; TLD – typical language development

### Inclusion criteria

Children with ASD were identified at specialized schools and had the diagnostic determined by a neurologist according to the DSM5 criteria. Children with DLD were contacted at a specialized university clinic and were assessed by SLPs an audiologist and a psychologist that determined the diagnosis based on the following criteria: delayed performance in language tests (mainly 1.5 SD bellow means in vocabulary and phonology tasks); cognitive and intellectual performance with standard score of 85 or higher; normal hearing (thresholds below 20 db) and no history of recent otitis media; no classical neurological symptoms and no facial deformities.

Children with TLD were contacted in regular schools and were paired with the children of the EG regarding age, type of school (private or public) and sex. Children with history of other disorders associated with language development were not included in this study. The information were obtained through the teachers and school records and later confirmed by the parents or caregivers, before the application of the questionnaire.

After this process of identification of the children, their parents or caregivers and teachers were contacted and invited to participate in the study. Extra inclusion criteria were that the caregivers had to be living with the child for at least four months and that the teachers worked with each child for at least 3 months. Therefore, the participants were those who attended to these criteria and agreed to participate in the study. All data was obtained before the COVID-19 pandemic; therefore, face-to-face contact was possible if necessary.

### Procedures

All participants answered the questionnaires of the “Assessment of Pragmatic Language and Social Communication – APLSC – parent and professional report – beta research version^(17)^ translated and adapted to Brazilian Portuguese by the last author of this study. The beta version of the tool aims to identify the type and quality of the communication and interaction of children between 3 and 6 years of age through a questionnaire answered by parents and/or caregivers and by professionals that interact routinely with the children, as the teachers. The questionnaire includes questions about pragmatic and social communication abilities. The answers are registered on a Likert format from 0 to 6 according to the frequency with which the behavior is observed (from “never” to “almost always”).

The questions related to the pragmatic aspects include information about the child’s active participation in dialogues, producing adequate comments and commenting their actions. They also question if the child is understood by other people and answers to other people. About social communicative abilities, the questions refer to the child’s participation in games and activities with other children and if the child engages in fights and disagreements.

In the original format the questionnaire to parents (with 30 questions) is different from the one to professionals (with 35 questions). To facilitate the analysis, only the questions that are included in both questionnaires were considered in this study.

The analysis of the questions considered four aspects: (1) “What are the different types of communicative functions used by small children?”; (2) “How many times are they used?”; (3) “Are the same communicative acts used at home and at the school?” And (4) “How are the answers to both questionnaires associated?”

The participants received instructions about the purpose of the questionnaire and how to answer it. The same procedure proposed by the authors was used to obtain the answers by the parents/caretakers: the questionnaires were sent home with the children along with an explanation letter. The teachers also received a verbal explanation by the researchers.

**Figure 1:**
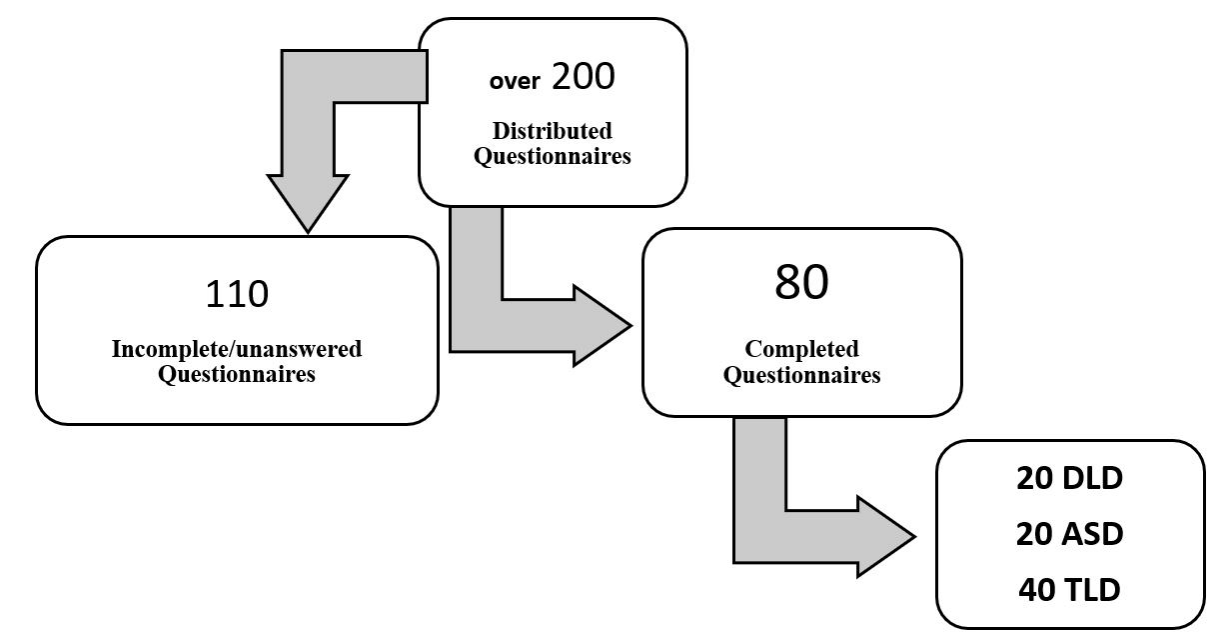
Chart-flow of the number of questionnaires/participants

The procedures of data gathering are synthesized in Figure 1.

Caption: ASD - Autism Spectrum Disorder; DLD - Developmental Language Disorder; TLD - Typical Language Development

Over 200 questionnaires were distributed but a large number of them were incomplete or unanswered.

### Analyses

Data were submitted to statistical analysis by the *One-Sample Kolmogorov-Smirnov Test* to verify if there was normal distribution. The results were considered significant when (p)≤ 0.05 and tendency values were considered when 0.05 ≤ (p) ≥ 0.10. The standard deviation was defined to each variable. Descriptive analysis used t-test and the Mann-Whitney test.

## RESULTS

Tables 1 and 2 describe the data and the comparison of the experimental groups ASD and DLD with their respective controls, considering the total scores on the questionnaire.

**Table 1:** Description and comparison of groups EGParentsASD and CGParentsTLD; and EGTeacherASD and CGTeacherTLD

**Variable**	**Group**		**n**	**Mean**	**Standard Deviation**	**MSE**	**Minimum**	**Maximum**	**Percentile 25**	**Percentile 50**	**Percentile 75**	**P Value**
Parents ASD	EG	10		47.00	26.94	8.52	8.00	92.00	29.25	43.00	68.50	0.01*
	CG	10		108.80	15.50	4.90	92.00	140.00	92.75	107.50	117.00	
Teacher ASD	EG	10		56.80	25.90	8.19	20.00	93.00	33.25	55.00	80.50	0.01*
	CG	10		103.70	8.42	2.66	88.00	114.00	98.00	107.00	109.00	

* -(p) value - significant difference (P<0.05)

**Caption:** n - number of questionnaires; EG - Experimental Group; CG - Comparative Group; MSE - Mean Standard Error

**Table 2:** Description and comparison of groups EGParentsDLD and CGParentsTLD; and EGTeacherDLD and CGTeacherTLD

**Variable**	**Group**		**n**	**Mean**		**Standard Deviation**		**MSE**		**Minimum**	**Maximum**	**Percentile 25**	**Percentile 50**	**Percentile 75**	**P Value**
Parents DLD		EG	10		99.70		18.60		5.88	55.00	123.00	91.00	105.00	109.75	0.67
		CG	10		105.30		11.42		3.61	89.00	121.00	93.75	106.50	115.25	
Teacher DLD		EG	10		82.00		26.49		8.37	40.00	123.00	59.00	84.00	105.00	0.10
		CG	10		102.10		22.52		7.12	60.00	144.00	91.00	103.50	111.25	

* -(p) value - significant difference (P<0.05)

**Caption:** n - number of questionnaires; EG - Experimental Group; CG - Comparative Group; MSE - Mean Standard Error

Table 3 describes the data and the comparison between the experimental groups (EGParentsASD - EGParentsDLD; EGTeacherASD-EGTeacherTLD).

**Table 3:** Description and comparison of groups EGParentsASD and EGParentsTLD; and EGTeacherASD and CGTeacherTLD

**Variable**	**Group**	**n**	**Mean**	**Standard Deviation**	**MSE**	**Minimum**	**Maximum**		**Percentile 25**	**Percentile 50**	**Percentile 75**	**P - Value**
Parents	EGDLD	10	99.70	18.60	5.88	55.00	123.00	91.00		105.00	109.75	0.01*
	EGASD	10	47.00	26.94	8.52	8.00	92.00	29.25		43.00	68.50	
Teacher	EGDLD	10	82.00	26.49	8.37	40.00	123.00	59.00		84.00	105.00	0.06
	EGASD	10	56.80	25.90	8.19	20.00	93.00	33.25		55.00	80.50	

* -(p) value - significant difference (P<0.05)

**Caption:** n - number of questionnaires; EG - Experimental Group; CG - Comparative Group; MSE - Mean Standard Error

It was observed that group EGParentsDLD had different results than group EGParentsASD (P < 0,05). The group EGTeacherDLD has shown a tendency to a higher score (82.00) than group EGTeacherASD (56.80) (p = 0.06).

Table 4 shows that there was no significant difference in the questionnaires answered by teachers.

**Table 4:** Comparison of the results of the questionnaires answered by teachers and parents of the children from the experimental (ASD and DLD) and the comparison groups (TLD)

**Variable**	**Group**	**n**	**Mean**	**Standard Deviation**	**MSE**	**Minimum**	**Maximum**	**t**	**df**	**P Value**
Parents/Teacher	ASD	20	-9.80	15.782	4.991	-21.090	1.490	-1.964	9	0.081
Parents/Teacher	DLD	20	17.70	24.958	7.892	-0.154	35.554	2.243	9	0.052
Parents/Teacher	TLD	40	4.15	19.610	4.385	-5.028	13.328	0.946	19	0.356

(t) null hypothesis

**Caption:** n - number of questionnaires; ASD - Autistic Spectrum Disorder; DLD - Developmental Language Disorder; TLD - Typical Language Development.; MSE - Mean Standard Error; df – degrees of freedom.

## DISCUSSION

The analysis refers to 80 questionnaires, which means that only 40% of all the 200 questionnaires that were distributed were returned. This apparently low rate of return is similar to that obtained by other researchers with the similar procedures^(15)^.

The comparison of the total scores of the questionnaires answered by parents and teachers of children with ASD and TLD has shown a significant difference - p<0.05 (Table 1). The mean score of the group of children with ASD was 47.00 when the questionnaires were answered by the parents, and 56.80 when the teachers answered the questionnaires. In what refers to the children with TLD the mean scores were 108.80 and 103.80 respectively. The minimum score attributed to a child with TLD was higher than the mean score attributed to the children with ASD. These differences were expected because interactive social and pragmatic impairments are systematically described in children with ASD^(5,18)^.

Discourse managing abilities, as the ability to adapt the language used to the needs of a conversational partner are part of the social communication and pragmatic impairments of persons with ASD^(19)^. Language comprehension and cultural competence are also poor, particularly in what refers to understanding and using language subtleties, humor, irony and the interpretation of non-verbal aspects of communication^(3,20)^.

Other authors suggest that difficulties with symbolic play, creativity and pragmatics may interfere with the patterns of social interaction and lead to less opportunities of social experiences^(16,19)^. Therefore, tests and assessment protocols, like the one used in this study, that include information about communicative initiative, conversational reciprocity, intonation, use and comprehension of gestures, prosody and facial expression in communication are essential to the identification of these difficulties.

In the comparison of the questionnaires answered by parents and teachers of children with DLD and with TLD, no significant differences were observed. The questionnaires answered by the parents (p=0.067) and teachers (0.10) of children of both groups presented similar scores (Table 2) but the mean, minimal and percentile results were higher to children with TLD children. The pragmatic profile of children with DLD received lower scores by parents and teachers in the items “spontaneously asks questions”, “is well understood in the first time that speaks”, “actively participates in conversation” and “explains his/her actions verbally”. Pragmatic disorders may be observed in children with DLD when there are structural language difficulties^(21)^. There is no doubt that the impairments in phonology, syntax and vocabulary that are frequently observed in DLD interfere with the pragmatic abilities^(22)^. However, in the present study these difficulties were not evident.

The conversational analysis of children with DLD shows that they present a larger number of inappropriate answers than children without language disorders and more difficulties to initiate communicative acts, demanding more time to engage in communicative situations^(23)^. The pragmatic difficulties interfere with the appropriate production and with the language comprehension in a given context. They include features as providing limited information to the conversational partner and inattention to social clues during a conversation. These aspects are included in the questionnaire that was analyzed in this study in questions such as if the child answers when questioned.

The answers of parents and teachers referring to children with ASD and DLD were also compared. When the answers by parents were considered we verified differences between the groups (Table 3, p=0.01) with a mean score of 99.70 to the group of children with ASD and 47.00 to the group of children with DLD. Considering the answers by teachers, data suggest a tendency of a similar difference between groups (Table 3 – p + 0.06). This tendency (and not difference) may be because teachers do not have a very positive perception about the communication of children with DLD. The structural language difficulties of children with DLD, such as inappropriately construed utterances, speech unintelligibility and use of non-specific vocabulary may have a higher impact in the use of language when the conversation includes more than one participant (reference). This may be more evident in the classroom context where there are several children participating in the conversations at the same time. The opposite is true in the case of teachers of children with ASD, that work only with children with restricted communication abilities, what may lead them to be less demanding about them^(19,21)^.

The comparison between parents’ and teachers’ scores to children of all the groups did not identify significant differences (Table 4). Scores attributed to children with TLD by parents and teachers were the most similar; therefore it is not possible to determine which of the respondent groups (parents or teachers) attributed scores closer to the children’s performance. Usually teachers and parents are excellent on assessing and observing the verbal responses and non-verbal communicative abilities of their students^(6,7,16)^ and can be important contributors to the speech-language assessment. Other authors^(22)^ interviewed parents and children about language difficulties of bilingual children and the answers provided by the teachers were closer to the children’s performance regarding receptive and expressive vocabulary.

The results of our study pointed out that children with ASD presented more severe impairments in social and pragmatic abilities than children with DLD. Also, the results indicate that both children with ASD and with DLD present poorer social and pragmatic abilities than children with TLD. Even children with DLD that are less efficient than their peers in the pragmatic aspects of communication, with less communication initiative and difficulties maintaining conversations^(23)^, seem to be able to present more efficient social performance, which places them on a higher level than their peers with ASD.

The association of the communicative impairments observed in children with ASD and DLD has been studied recently. Children and adolescents with DLD have a higher risk of presenting ASD characteristics. Prior studies^(24)^ observed that children that were diagnosed as having DLD at age 7, presented higher prevalence of autism at 14 than would be expected in the general population. The authors suggest that continuous experiences with language difficulties may be responsible for the symptoms of autism during adolescence. Therefore, identifying social and pragmatic difficulties in children with DLD during early childhood may help avoid that language disorders resulting in social and adaptive difficulties later in life.

## CONCLUSION

The assessment tool was useful in identifying the differences in performance of children with diverse social communicative profiles. Children with ASD presented social and pragmatic impairments that were more significant than those presented by children with DLD. However, both children with ASD and with DLD presented more social pragmatic difficulties than children with TLD. It was also possible to verify that the perception of parents and teachers regarding the social pragmatic abilities of children they are familiar with is very similar, regardless of the diagnosis.

Although the difference between children with DLD and TLD was not statistically significant, it can have clinical relevance since they may be associated with functional communication difficulties in everyday life and with school adaptation.

The small sample and the fact that it is limited to just one social and cultural group are the major limitations of this study. It would be important to obtain similar data on a larger and more diverse social/cultural group.
